# Distinct employment interference profiles in patients with breast cancer prior to and for 12 months following surgery

**DOI:** 10.1186/s12885-021-08583-0

**Published:** 2021-08-02

**Authors:** Raymond Javan Chan, Bruce Cooper, Louisa Gordon, Nicolas Hart, Chia Jie Tan, Bogda Koczwara, Kord M. Kober, Alexandre Chan, Yvette P. Conley, Steven M. Paul, Christine Miaskowski

**Affiliations:** 1 School of Nursing, Queensland University of Technology, Kelvin Grove, Australia; 2 Cancer and Palliative Care Outcomes Centre, Queensland University of Technology, Kelvin Grove, QLD Australia; 3 Princess Alexandra Hospital, Metro South Hospital and Health Services, Woolloongabba, Queensland Australia; 4 Department of Physiological Nursing, School of Nursing, University of California, San Francisco, 2 Koret Way – N631Y, San Francisco, CA 94143-0610 USA; 5 QIMR Berghofer Medical Research Institute, Brisbane, Queensland Australia; 6 Department of Pharmacy, Faculty of Science, National University of Singapore, Singapore, Singapore; 7 Department of Pharmacy, National Cancer Centre, Singapore, Singapore, Singapore; 8 Flinders Centre for Innovation in Cancer, Flinders Medical Centre and Flinders University, Bedford Park, Australia; 9 Department of Clinical Pharmacy Practice, University of California, Irvine, CA USA; 10 School of Nursing, University of Pittsburgh, Pittsburgh, PA USA

**Keywords:** Female breast cancer, Employment interference, Fatigue, Patient-reported outcomes, Quality of life, Sleep disturbance

## Abstract

**Purpose:**

To identify subgroups of female breast cancer patients with distinct self-reported employment interference (EI) profiles and determine which demographic, clinical, and symptom characteristics, and quality of life outcomes were associated with subgroup membership.

**Methods:**

Women with breast cancer (*n* = 385) were assessed for changes in EI over ten times, from prior to, through 12 months after breast cancer surgery. Latent profile analysis (LPA) was used to identify subgroups of patients with distinct EI profiles.

**Results:**

Three distinct EI profiles (i.e., None – 26.2% (*n* = 101), Low – 42.6% (*n* = 164), High – 31.2% (*n* = 120)) were identified. Compared to the None and Low groups, patients in the High group were more likely to be younger. Higher proportions in the High group were non-White, pre-menopausal prior to surgery, had more advanced stage disease, had received an axillary lymph node dissection, had received neoadjuvant chemotherapy, had received adjuvant chemotherapy, and had a re-excision or mastectomy on the affected breast within 6 months after surgery. In addition, these patients had lower quality of life scores. Compared to the None group, the High group had higher levels of trait and state anxiety, depressive symptoms, fatigue and sleep disturbance and lower levels of cognitive function.

**Conclusions:**

This study provides new knowledge regarding EI profiles among women in the year following breast cancer surgery. The non-modifiable risk factors (e.g., younger age, being non-White, having more advanced stage disease) can inform current screening procedures. The potentially modifiable risk factors can be used to develop interventions to improve employment outcomes of breast cancer patients.

**Supplementary Information:**

The online version contains supplementary material available at 10.1186/s12885-021-08583-0.

## Introduction

In 2020, over 270,000 new cases of female breast cancer were diagnosed in the United States [1]. A breast cancer diagnosis and its treatment have significant short- and long- term impacts on patients’ employment [2]. Depending on an individual’s situation, breast cancer patients report varying levels of absenteeism from work, reduced work ability, limited work performance and career progression, and at times, termination of employment [2, 3]. In addition, compared to men, women are in relatively less stable occupations, as well as in lower income and more casual employment, that results in more acute employment interference (EI) from their cancer [4]. The ability to return to work and to satisfactorily engage in work contributes to continued insurance benefits, financial well-being, sense of identity and accomplishment, and a return to a resemblance of normal [5].

The impacts of cancer on employment is a global issue [2]. In a Swedish cross sectional study of 756 women with breast cancer, 56% were still on sick leave at 4 to 6 weeks after surgery [6]. In a French study [7], over 80% of breast cancer survivors who were in paid work prior to their diagnosis had a median sick leave duration of 10.8 months. Older age, lower educational level, receipt of chemotherapy (CTX) or radiotherapy, lymphoedema, and insufficient support from employers or colleagues were associated with a delay in return to work [7]. For women who returned to work, diminished performance was associated with pain [8], anxiety [9–13], fatigue [9–13], depression [9–13], and cognitive dysfunction [10, 13, 14]. These limitations affected productivity, duration of the work day, and/or changes in work roles and responsibilities [5].

In the survivorship literature, most studies have focused on how the aforementioned factors affect return to work as a binary outcome [2, 15]. This binary outcome captures only extreme consequences, namely: no loss or complete loss of employment. In contrast, an assessment of the overall extent of employment interference (EI) has the advantage of evaluating the spectrum of potential negative outcomes. This approach may provide a more accurate picture of the impact of cancer on work and employment. Prior to our recent publication [16], no studies had examined patients’ subjective experiences with the extent of EI, as well as changes in their experiences over time and associated predictors. In our recent longitudinal study [16], we used hierarchical linear modeling (HLM) to evaluate for inter-individual differences in self-reported EI in 387 women with breast cancer over 12 months. In this study [16], factors associated with higher levels of self-reported EI were younger age, a lower annual household income, higher pain intensity, higher sleep disturbance, and the receipt of an axillary lymph node dissection (ALND), a sentinel lymph node biopsy (SLNB), adjuvant CTX, complementary or alternative therapies, or a re-excision or mastectomy.

While our previous study [16] provides some insights into the characteristics associated with EI in patients with breast cancer, the statistical approach used did not allow for the identification of subgroups of patients with distinct EI profiles. The use of person-centered analytic approaches, like latent profile analysis (LPA), allows for the identification of these subgroups, as well as for the identification of demographic and clinical characteristics associated with subgroup membership. This type of analysis can provide information on high risk patients who can be targeted for appropriate interventions. Therefore, the purposes of this study were to use LPA to identify subgroups of breast cancer patients with distinct self-reported EI profiles and to determine which demographic, clinical, and symptom characteristics, as well as quality of life (QOL) outcomes, were associated with each subgroup. In order to be able to determine risk factors for EI, all of the patients in the sample were included regardless of their employment status at enrollment.

## Methods

### Patients and settings

The methods used in this analysis are described in detail in previous publications [17–20] and are summarized in this methods section. This descriptive, longitudinal analysis is part of a larger National Cancer Institute-funded study that evaluated for neuropathic pain and lymphedema in women who underwent breast cancer surgery [17–19]. Patients were recruited from Breast Care Centers located in a Comprehensive Cancer Center, two public hospitals, and four community practices in Northern California. Patients were eligible to participate if they were women > 18 years of age who would undergo breast cancer surgery on one breast; were able to read, write, and understand English; agreed to participate; and gave written informed consent. Patients were excluded if they were having breast cancer surgery on both breasts and/or had distant metastases at the time of diagnosis.

### Study procedures

The study was approved by the Committee on Human Research at the University of California, San Francisco and by the Institutional Review Board at each of the study sites. During the patient’s preoperative visit, a clinical staff member explained the study to the patient, determined her willingness to participate, and introduced her to the research nurse. The research nurse met with the women, determined eligibility, and obtained written informed consent prior to surgery. After obtaining consent, patients completed the enrollment questionnaires an average of 4 days prior to surgery and at one, two, three, four, five, six, eight, 10, and 12 months after surgery. The research nurse met with the patients in the Clinical Research Center or in their homes. Patients’ medical records were reviewed for disease and treatment information.

### Instruments

At enrollment, patients completed a demographic questionnaire, the Karnofsky Performance Status (KPS) scale [21], and the Self-Administered Comorbidity Questionnaire (SCQ) [22]. The KPS scale, that ranged from 40 (disabled, requires special care and assistance) to 100 (normal, no complaints; no evidence of recurrence) was used to evaluate functional status [21]. The SCQ evaluates the occurrence of, treatment for, and impact of 13 common medical conditions. The total SCQ score ranges from 0 to 39 [22].

For this study, EI was assessed using a single item from the Quality of Life Scale-Patient Version (QOL-PV) [23], namely, “To what degree has your illness or treatment interfered with your employment?”. Patients were asked to rate this item using a 0 (no problem) to 10 (severe problem) numeric rating scale (NRS). No gold standard exists to measure employment outcomes [2]. Our approach allowed us to assess patients’ subjective perceptions of the overall impact of cancer and its treatment on their employment [16]. Another advantage of the evaluation of self-reported EI with a single item rather than return to work (yes/no) is that the latter outcome is rather simplistic and does not take into account external factors that are unrelated to cancer but may influence employment, such as aging and changes in outlook on life [5, 24]. The phrasing of the EI item has the advantage of having the patient focus on how their employment was disrupted specifically by cancer and its treatment.

The Spielberger State-Trait Anxiety Inventories (STAI-S, STAI-T) were used to assess the patient’s transitory emotional response to a stressful situation and her predisposition to anxiety, respectively. Scores for each scale are summed and can range from 20 to 80. Higher scores indicate greater anxiety. Cut-off scores of > 31.8 and > 32.2 indicate high levels of trait and state anxiety, respectively [25]. Both inventories have well-established validity and reliability [26, 27]. In this study, Cronbach’s alphas for the STAI-T and STAI-S were .88 and .95, respectively.

The 20-item Center for Epidemiological Studies Depression Scale (CES-D) was used to evaluate the major symptoms in the clinical syndrome of depression. Scores can range from 0 to 60, with scores of > 16 indicating the need for individuals to seek clinical evaluation. The CES-D has well-established validity and reliability [28–30]. In this study, its Cronbach’s alpha was 0.90.

The 18-item Lee Fatigue Scale (LFS) was used to assess physical fatigue and energy [31]. Each item was rated on a 0 to 10 NRS. Total fatigue and energy scores were calculated as the mean of the 13 fatigue and the five energy items. Higher scores indicate greater fatigue severity and higher levels of energy. Patients were asked to rate each item based on how they felt “right now”. Cut-off scores of > 4.4 and < 4.8 indicate high levels of fatigue and low levels of energy, respectively [32]. The LFS has well established validity and reliability [31]. In this study, Cronbach’s alphas for fatigue and energy scales were .96 and .93, respectively.

The 16-item Attentional Function Index (AFI) was used to measure attentional function [33]. Each item was rated on a 0 to 10 NRS. A higher mean score indicates greater capacity to direct attention [33, 34]. Scores are grouped into categories of attentional function (i.e., < 5.0 low function, 5.0 to 7.5 moderate function, > 7.5 high function) [35]. The AFI has well established validity and reliability [34, 36]. In this study, its Cronbach’s alpha was .95.

The 21-item General Sleep Disturbance Scale (GSDS) was used to assess the quality of sleep in the past week. Each item was rated on a 0 (never) to 7 (everyday) NRS. The GSDS total score is the sum of 21 items that can range from 0 (no disturbance) to 147 (extreme sleep disturbance). A GSDS total score of > 43 indicates a significant level of sleep disturbance [37]. The GSDS has well established validity and reliability [38, 39]. In this study, its Cronbach’s alpha was .86.

The occurrence of breast pain prior to surgery was determined by asking the question “Are you experiencing pain in your affected breast?” If women responded yes, they rated their average and worst pain using a 0 (no pain) to 10 (worst imaginable pain) NRS.

The 41-item QOL-PV was used to assess four domains of QOL (i.e., physical well-being, psychological well-being, social well-being, spiritual well-being). Items are rated on a 0 to 10 NRS. Mean subscale and total scores were calculated. Higher scores indicate better QOL. The QOL-PV has well established validity and reliability [40, 41]. Cronbach’s alphas for the QOL-PV physical well-being, psychological well-being, social well-being, and spiritual well-being subscales, as well as the total QOL scale, were: .80, .86, .80, .63, and .86, respectively.

### Data analysis

Descriptive statistics and frequency distributions were computed for sample characteristics, symptom severity scores, and QOL scores using SPSS version 27 (IBM Corporation, Armonk, NY). As previously described [42], unconditional LPA was used to identify the profiles of self-reported EI that characterized unobserved subgroups (i.e., latent classes) of patients over the 12 months of the study. First, all of the patients who reported a zero for the EI item across the 10 assessments were categorized in the None group. Then, we identified subgroups of patients based on their profiles of means across the 10 assessments for the EI item from the QOL-PV. In order to incorporate the expected correlations among the repeated measures, we included covariance among the EI scores that were up to four occasions apart (i.e., a covariance structure with a lag of four). In this way, we retained the within-person correlation among the self-reported EI scores, while we focused on the patterns of means that distinguished among the latent classes. We limited the covariance structure to a lag of four to accommodate the expected reduction in correlation that would be introduced by decreased stability in the EI ratings as the separation of months increased and to reduce model complexity.

Estimation was carried out with full information maximum likelihood with standard errors and a Chi-square test that are robust to non-normality and non-independence of observations (“estimator = MLR”). Model fit was evaluated to identify the best solution that characterized the observed latent class structure with the Bayesian Information Criterion (BIC), the Vuong-Lo-Mendell-Rubin (VLMR) likelihood ratio test the K vs. K^− 1^ model, entropy, and latent class percentages that were large enough to be reliable (i.e., likely to replicate in new samples) [43]. Missing data were accommodated with the use of the Expectation-Maximization algorithm [44]. Mixture models, like LPA, are known to produce solutions at local maxima. Therefore, our models were fit with from 800 to 1600 random starts. This approach ensured that the estimated model was replicated many times and was not due to a local maximum. Estimation was done with Mplus Version 7.2 [45].

After identifying the latent class solution that best fit the data, differences among EI groups, in demographic and clinical characteristics, symptom scores, and QOL scores, obtained at enrollment, were evaluated using analyses of variance, Chi-square, and Kruskal-Wallis analyses. A *p*-value of < 0.05 was considered statistically significant. Post-hoc contrasts were done using a Bonferroni corrected *p*-value of < 0.017 (0.05/3 pairwise comparisons).

## Results

### Latent profile analysis

**Fig. 1:**
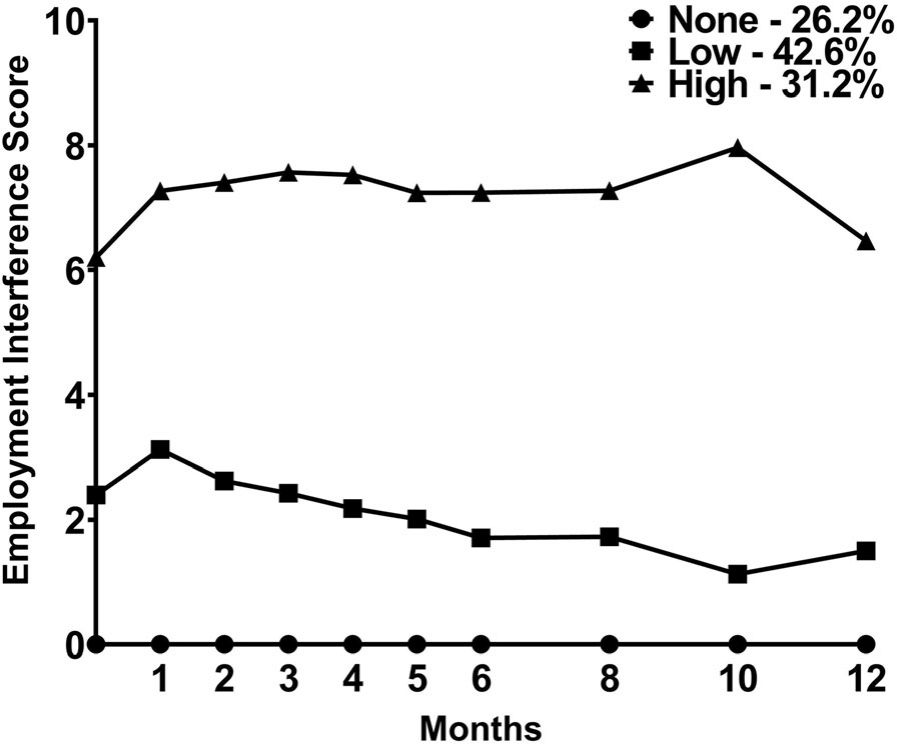
Employment interference trajectories for patients in each of the latent classes

Data from 385 patients with breast cancer were used in the LPA. As shown in Fig. 1, 26.2% of patients (*n* = 101) did not report any EI for all of the assessments and were named the None class. Among the remaining 284 patients, two distinct latent classes were identified. A two-class model was selected because its BIC was lower than the BIC for the 1-class solution (Table 1). The VLMR was statistically significant for the 2-class solution, indicating that two classes fit the data better than one class. In addition, the VLMR was not significant for the 3-class solution, indicating that too many classes were extracted.

**Table 1:** Employment Interference: Latent Profile Solutions and Fit Indices for One- Through Three-Class Solutions

Model	LL	AIC	BIC	Entropy	VLMR
1 Class	− 5115.38	10,318.75	10,479.30	n/a	n/a
2 Class	− 4937.42	9982.84	10,179.88	.92	355.91^+^
3 Class	− 4885.99	9899.98	10,133.52	.87	102.85^ns^

^+^*p* < .0001

*Abbreviations*: *AIC* Akaike’s Information Criterion, *BIC* Bayesian Information Criterion, *LL* log-likelihood, *n/a* not applicable for one class, *ns* not significant, *VLMR* Vuong-Lo-Mendell-Rubin likelihood ratio test for the K vs. K-1 model

As shown in Fig. 1, the largest proportion of the patients were classified in the Low class (*n* = 164, 42.6%). This group reported a mean EI score of 2.4 (+ 2.9) at enrollment, that increased slightly at month 1 and then decreased over the 12 months of the study. The second class that consisted of 31.2% (*n* = 120) of the women had a mean EI score of 6.2 (+ 3.5) at enrollment and was named the High class. This group’s EI scores were in the moderate range at enrollment and increased and remained in the moderate to high range in the 12 months following surgery.

### Differences in demographic and clinical characteristics

As shown in Table 2, no differences were found among the three groups in body mass index, marital status, living arrangements, number of hours worked per week (for those who were employed), time since diagnosis, type of surgery, receipt of SLNB, receipt of breast reconstruction at the time of surgery, or pain in the affected breast. In addition, no differences were found among the three groups in the receipt of radiation therapy, hormonal therapy, or breast reconstruction in the 12 months following surgery.

**Table 2:** Differences in demographic and clinical characteristics among the employment interference latent classes at enrollment

Characteristic	None (0) 26.2% (*n* = 101)	Low (1) 42.6% (*n* = 164)	High (2) 31.2% (*n* = 120)	Statistics
	Mean (SD)	Mean (SD)	Mean (SD)	
Age (years)	62.4 (11.4)	54.8 (10.3)	48.7 (9.5)	*F =* 48.04, *p* < 0.001 0 > 1 > 2
Education (years)	15.0 (2.6)	16.1 (2.5)	15.9 (2.7)	*F =* 5.46, *p* = 0.005 0 < 1 and 2
Self-Administered Comorbidity Questionnaire score	4.8 (2.9)	3.8 (2.8)	4.3 (2.7)	*F =* 3.90, *p* = 0.021 0 > 1
Karnofsky Performance Status score	95.7 (8.0)	93.2 (10.7)	91.3 (11.0)	*F =* 5.17, *p* = 0.006 0 > 2
Body mass index (kg/m^2^)	27.5 (6.7)	26.1 (5.6)	27.4 (6.2)	*F =* 2.46, *p* = 0.086
	% (n)	% (n)	% (n)	
Ethnicity – Non-White	30.7 (31)	26.2 (43)	47.5 (57)	X^2^ = 14.66, *p =* 0.001 0 and 1 < 2
Married/partnered (% yes)	41.6 (42)	38.4 (63)	45.0 (54)	X^2^ = 1.24, *p* = 0.537
Lives alone (% yes)	27.7 (28)	22.6 (37)	20.8 (25)	X^2^ = 1.56, *p* = 0.459
Currently employed (% yes)	16.8 (17)	68.9 (113)	48.3 (58)	X^2^ = 67.85, *p <* 0.001 0 < 1 and 2, 1 > 2
Number of work hours per week (% yes)				X^2^ = 1.43, *p* = 0.838
1–35 h/week	38.9 (7)	36.3 (41)	44.8 (26)	
36–44 h/week	44.4 (8)	48.7 (55)	39.7 (23)	
45h hours/week	16.7 (3)	15.0 (17)	15.5 (9)	
Annual household income				KW, *p <* 0.001 0 < 1, 1 > 2
< $30,000^a^	23.1 (18)	10.3 (15)	34.4 (33)	
$30,000 to <$70,000	28.2 (22)	24.0 (35)	25.0 (24)	
$70,000 to <$100,000	14.1 (11)	20.5 (30)	10.4 (10)	
> $100,000	34.6 (27)	45.2 (66)	30.2 (29)	
Gone through menopause prior to surgery (% yes)	79.2 (80)	65.9 (108)	50.8 (61)	X^2^ = 19.50, *p <* 0.001 0 and 1 > 2
Days since cancer diagnosis (mean (SD))	57.3 (63.9)	67.7 (83.7)	84.8 (77.0)	KW, *p* = 0.079
Days since cancer diagnosis (median)	39.0	36.0	40.50	
Stage of disease				KW, *p <* 0.001 0 and 1 < 2
Stage 0	16.8 (17)	23.8 (39)	14.2 (17)	
Stage I	53.5 (54)	37.2 (61)	26.7 (32)	
Stage IIa and IIb	25.7 (26)	32.9 (54)	45.8 (55)	
Stage IIIa, IIIb, IIIc, and IV^a^	4.0 (4)	6.1 (10)	13.3 (16)	
Type of surgery				X^2^ = 6.84, *p* = 0.033 No significant post hoc contrasts
Breast conservation	85.1 (86)	74.4 (122)	85.0 (102)	
Mastectomy	14.9 (15)	25.6 (42)	15.0 (18)	
Sentinel lymph node biopsy (% yes)	84.2 (85)	84.8 (139)	79.2 (95)	X^2^ = 1.69, *p* = 0.430
Axillary lymph node dissection (% yes)	23.8 (24)	31.7 (52)	54.2 (65)	X^2^ = 24.82, *p <* 0.001 0 and 1 < 2
Underwent reconstruction at the time of surgery (% yes)	14.9 (15)	25.0 (41)	23.3 (28)	X^2^ = 4.01, *p* = 0.135
Received neoadjuvant therapy (% yes)	11.9 (12)	17.7 (29)	30.8 (37)	X^2^ = 13.37, *p =* 0.001 0 and 1 < 2
Pain in the affected breast prior to surgery (% yes)	20.8 (21)	27.4 (45)	31.7 (38)	X^2^ = 3.32, *p* = 0.191
Received radiation therapy during the 12 months following surgery (% yes)	68.3 (69)	70.1 (115)	75.0 (90)	X^2^ = 1.35, *p* = 0.510
Received chemotherapy during the 12 months following surgery (% yes)	26.7 (27)	29.9 (49)	45.8 (55)	X^2^ = 11.10, *p* = 0.004 0 and 1 < 2
Received hormonal therapy during the 12 months following surgery (% yes)	64.4 (65)	61.0 (100)	55.8 (67)	X^2^ = 1.73, *p* = 0.422
Received complementary therapy in the 12 months following surgery (% yes)	19.8 (20)	39.6 (65)	36.7 (44)	X^2^ = 11.82, *p* = 0.003 0 < 1 and 2
Had breast reconstruction in the 12 months following surgery (% yes)	7.9 (8)	14.6 (24)	11.7 (14)	X^2^ = 2.69, *p* = 0.260
Had re-excision or mastectomy on the affected breast in the six months following surgery (% yes)	24.8 (25)	25.6 (42)	41.7 (50)	X^2^ = 10.50, *p =* 0.005 0 and 1 < 2

^a^reference group

*Abbreviations*: *kg* kilograms, *KW* Kruskal-Wallis, *m*^*2*^ meters squared, *SD* standard deviation

Compared to the other two groups, patients in the High group were more likely to be younger, non-White, pre-menopausal prior to surgery, had more advanced stage disease, and had received an ALND, neoadjuvant CTX, adjuvant CTX, and a re-excision or mastectomy on the affected breast within 12 months after surgery. Compared to the Low group, patients in the High group were less likely to be employed and had a lower annual household income prior to surgery. Compared to the None group, patients in the Low group were more likely to be younger, had a lower comorbidity score, and had a higher annual household income. Compared to the None group, patients in the other two groups had more years of education, were more likely to be employed prior to surgery, and more likely to use complementary therapy in the 12 months following surgery.

### Differences in symptom severity scores

As shown in Table 3, based on their responses at enrollment, no differences were found among the three groups in energy or pain scores prior to surgery. Compared to the None group, the High group had higher scores for trait and state anxiety, depressive symptoms, fatigue, and sleep disturbance and lower scores for attentional function. Compared to the None group, the Low group had higher fatigue scores at enrollment.

**Table 3:** Differences in symptom severity scores among the employment interference latent classes at enrollment

Symptom^a^	None (0) 26.2% (*n* = 101)	Low (1) 42.6% (*n* = 164)	High (2) 31.2% (*n* = 120)	Statistics
	Mean (SD)	Mean (SD)	Mean (SD)	
Trait anxiety score (> 31.8)	33.7 (8.6)	35.1 (8.4)	36.7 (9.5)	*F =* 3.20, *p* = 0.042 0 < 2
State anxiety score (> 32.2)	38.7 (13.4)	41.7 (13.0)	43.3 (13.0)	*F =* 3.52, *p* = 0.031 0 < 2
Center of Epidemiologic Studies-Depression Scale score (> 16.0)	11.2 (9.3)	13.8 (9.2)	15.3 (10.4)	*F =* 5.01, *p* = 0.007 0 < 2
Lee Fatigue Scale – fatigue score (> 4.4)	2.3 (2.0)	3.2 (2.2)	3.6 (2.6)	*F =* 9.32, *p <* 0.001 0 < 1 and 2
Lee Fatigue Scale – energy score (< 4.8)	5.1 (2.8)	4.9 (2.2)	4.7 (2.5)	*F =* 0.58, *p* = 0.558
Attentional Function Index score (< 5.0 is low, 5.0 to 7.5 is moderate, > 7.5 is high)	7.1 (2.0)	6.6 (1.8)	6.1 (2.0)	*F =* 7.06, *p* = 0.001 0 > 2
General Sleep Disturbance Scale score (> 43.0)	43.7 (21.9)	48.0 (21.3)	51.8 (20.4)	*F =* 4.06, *p* = 0.018 0 < 2
Average pain intensity score in the breast prior to surgery	0.5 (1.5)	0.5 (1.1)	0.7 (1.5)	KW, *p* = 0.293
Worst pain intensity score in the breast prior to surgery	0.6 (1.6)	0.9 (1.7)	1.2 (2.3)	KW = 0.200

^a^Clinically meaningful cutpoints for symptom severity are in parentheses

*Abbreviations*: *KW* Kruskal-Wallis, *SD* standard deviation

### Differences in QOL scores

As shown in Table 4, based on their responses at enrollment, significant differences were found among the three groups in psychological well-being, social well-being, and total QOL scores (None > Low > High). Compared to the other two groups, patients in the High group had lower physical well-being scores. Compared to the Low group, the High group had higher spiritual well-being scores.

**Table 4:** Differences in quality of life scores among the employment interference latent classes at enrollment

Quality of Life	None (0) 26.2% (*n =* 101)	Low (1) 42.6% (*n* = 164)	High (2) 31.2% (*n* = 120)	Statistics
	Mean (SD)	Mean (SD)	Mean (SD)	
Physical well-being	8.5 (1.4)	8.2 (1.4)	7.3 (1.8)	*F =* 19.05, *p <* 0.001 0 and 1 > 2
Psychological well being	6.7 (1.8)	5.7 (1.7)	5.1 (1.7)	*F =* 20.39, *p <* 0.001 0 > 1 > 2
Social well-being	8.3 (1.3)	7.2 (1.6)	5.5 (2.0)	*F =* 69.95, *p <* 0.001 0 > 1 > 2
Spiritual well-being	5.6 (1.8)	5.5 (1.7)	6.1 (2.0)	*F =* 3.84, *p* = 0.022 1 < 2
Total quality of life score	7.2 (1.2)	6.5 (1.2)	5.8 (1.4)	*F =* 29.45, *p <* 0.001 0 > 1 > 2

*Abbreviation*: *SD* standard deviation

## Discussion

This longitudinal study is the first to use LPA to identify subgroups of patients with distinct self-reported EI profiles in a large sample of women who were assessed prior to and for 12 months following breast cancer surgery. Cancer- and treatment-related EI is an important issue for women, with 42.6% reporting relatively low levels of EI and 31.2% reporting relatively high levels of EI over the 12 months after surgery. While direct comparisons are difficult because of differences in study measures, our findings are consistent with previous research that highlighted the high prevalence of EI in these patients [2, 5].

Rather than only selecting women who were employed at the pre-operative assessment, our approach of including all women regardless of current employment status allowed us to determine risk factors for EI in the entire breast cancer patient cohort. There are two advantages of using this approach. First, unemployment at the pre-surgical assessment is not necessarily permanent. That is, women who were unemployed could still have the capacity and desire to engage in paid work over the course and after the completion of their treatment. Second, the risk factors identified in this study apply to all women receiving surgery and are applicable for the identification of high risk patients in the clinic. While only 48.8% of these women (*n* = 188) reported “currently working for pay” at enrollment, the assessments of EI identified that 73.8% of our patients (*n* = 284) experienced some level of EI over the 12-month period. This discrepancy may be due to changes in women’s employment status before and following surgery. While these findings support our approach of including the entire sample of women in our analysis, they suggest that in future studies, investigators should evaluate employment status, intent to seek employment, and EI at multiple time points following surgery.

Compared to our previous HLM analysis of EI [16], the current LPA identified additional risk factors in those women who experienced high levels of EI (see Additional file 1). In terms of additional non-modifiable risk factors, compared to women with no EI, patients with high levels of EI had a higher level of education; were more likely to be non-White and pre-menopausal prior to surgery; had more advanced stage disease; and had received neoadjuvant CTX. These risk factors are known to impact employment [46]. While findings from a meta-analysis of previous studies found that lower levels of education were associated with an increased level of unemployment following a cancer diagnosis [46], we found that compared to patients with no EI, women in the other two EI groups had more years of education. This inconsistent finding may be related to the older age of the women in the No EI group and that a higher proportion of the patients in this group were retired or working part-time. In contrast, given that the women in the other two groups were younger and better educated, they may have had more demanding jobs and a higher expectation to return to work [5, 46]. Consistent with the literature, a non-White background [3, 47], receipt of neoadjuvant CTX [46], and having more advanced disease [46] were associated with worse employment outcomes in patients with breast cancer.

Consistent with the findings from a systematic review [48], our study highlights a number of potentially modifiable risk factors associated with EI, including a lower functional status, lower levels of energy and cognitive function, as well as higher levels of trait and state anxiety, depressive symptoms, sleep disturbance, and fatigue. While the clear association between a higher symptom burden and poorer work outcomes is not surprising, our findings provide additional evidence of the need to systematically incorporate symptom management interventions in return to work plans. According to a Cochrane review [49], while current return to work interventions may have one or a combination of physical, psychosocial and vocation components, they do not consistently incorporate a symptom management focus. Our findings suggest that the management of the modifiable risk factors including unrelieved symptoms in these interventions may facilitate a patient’s ability to return to work with lower levels of EI. Recently, Alfano and colleagues called for a personalized, tailored approach to address factors that affect cancer survivors’ ability to work during and/or after treatment [50]. To enhance the personalization of interventions, additional research is warranted to determine the most appropriate combination of interventions, based on individual risk factors [50].

Consistent with findings from a systematic review [51], lower levels of EI were associated with better physical, psychological, and social well-being. Of note, fewer studies have examined the relationships between spiritual well-being and work outcomes in cancer survivors [51]. Similar to our previous report on the relationships between financial toxicity and quality of life [20], patients with high levels of EI reported higher levels of spiritual well-being. This finding may be partially explained by the higher proportion of Non-white (47.5%) patients in the High group. Non-white patients are more likely to report higher levels of spiritual well-being [52], especially among African Americans who have a religious and church affiliation [53].

This study has some limitations. First, because no single gold standard is available to measure employment outcomes, EI was measured using a single item. This approach did not allow for a comprehensive assessment of all relevant aspects of work ability and performance (e.g., work satisfaction, time off work, work quality). While our single item is valid and reliable, future studies should perform a more detailed evaluation of EI. In addition, this study was conducted in Breast Care Centers in the United States, which limits the generalizability of the findings to other countries where women may have different experiences with EI. This study was conducted pre-COVID-19. Future studies should investigate how EI profiles and associated factors were or are being impacted by the pandemic.

In conclusion, this longitudinal study with 12 months of follow-up provides new knowledge about distinct subgroups of women with varying levels of EI during and following breast cancer surgery. The non-modifiable and potentially modifiable risk factors associated with EI can assist clinicians to identify high risk patients. The risk factors associated with the High and Low groups can be incorporated into current screening procedures and/or treatment algorithms that would facilitate referrals for multidisciplinary return-to-work and symptom management interventions.

## Supplementary Information

**Additional file 1.**

## Data Availability

The datasets used and/or analysed during the current study are available from the corresponding author on reasonable request.

## Abbreviations

AFI: Attentional Function Index
ALND: Axillary lymph node dissection
CES-D: Center for Epidemiological Studies Depression Scale
CTX: Chemotherapy
EI: Employment interference
GSDS: General Sleep Disturbance Scale
HLM: Hierarchical linear modeling
LFS: Lee Fatigue Scale
LPA: Latent profile analysis
NRS: Numeric rating scale
QOL: Quality of life
SLNB: Sentinel lymph node biopsy
STAI: Spielberger State-Trait Anxiety Inventories

## References

[1] Siegel RL, Miller KD, Jemal A (2020). Cancer statistics, 2020. CA Cancer J Clin.

[2] Sun Y, Shigaki CL, Armer JM (2017). Return to work among breast cancer survivors: a literature review. Support Care Cancer.

[3] Jagsi R, Abrahamse PH, Lee KL, Wallner LP, Janz NK, Hamilton AS, Ward KC, Morrow M, Kurian AW, Friese CR, Hawley ST, Katz SJ (2017). Treatment decisions and employment of breast cancer patients: results of a population-based survey. Cancer.

[4] Bradley CJ, Neumark D, Luo Z, Schenk M (2007). Employment and cancer: findings from a longitudinal study of breast and prostate cancer survivors. Cancer Investig.

[5] Butow P, Laidsaar-Powell R, Konings S, Lim CYS, Koczwara B (2020). Return to work after a cancer diagnosis: a meta-review of reviews and a meta-synthesis of recent qualitative studies. J Cancer Surviv.

[6] Petersson LM, Wennman-Larsen A, Nilsson M, Olsson M, Alexanderson K (2011). Work situation and sickness absence in the initial period after breast cancer surgery. Acta Oncol.

[7] Fantoni SQ, Peugniez C, Duhamel A, Skrzypczak J, Frimat P, Leroyer A (2010). Factors related to return to work by women with breast cancer in northern France. J Occup Rehabil.

[8] Quinlan E, Thomas-MacLean R, Hack T, Kwan W, Miedema B, Tatemichi S, Towers A, Tilley A (2009). The impact of breast cancer among Canadian women: disability and productivity. Work.

[9] Ahn E, Cho J, Shin DW, Park BW, Ahn SH, Noh DY, Nam SJ, Lee ES, Yun YH (2009). Impact of breast cancer diagnosis and treatment on work-related life and factors affecting them. Breast Cancer Res Treat.

[10] Calvio L, Peugeot M, Bruns GL, Todd BL, Feuerstein M (2010). Measures of cognitive function and work in occupationally active breast cancer survivors. J Occup Environ Med.

[11] Carlsen K, Jensen AJ, Rugulies R, Christensen J, Bidstrup PE, Johansen C, Huitfeldt Madsen IE, Dalton SO (2013). Self-reported work ability in long-term breast cancer survivors. A population-based questionnaire study in Denmark. Acta Oncol.

[12] Hansen JA, Feuerstein M, Calvio LC, Olsen CH (2008). Breast cancer survivors at work. J Occup Environ Med.

[13] Todd BL, Feuerstein EL, Feuerstein M (2011). When breast cancer survivors report cognitive problems at work. Int J Psychiatry Med.

[14] Breckenridge LM, Bruns GL, Todd BL, Feuerstein M (2012). Cognitive limitations associated with tamoxifen and aromatase inhibitors in employed breast cancer survivors. Psychooncology.

[15] Cocchiara RA, Sciarra I, D'Egidio V, Sestili C, Mancino M, Backhaus I, Mannocci A, De Luca A, Frusone F, Di Bella O (2018). Returning to work after breast cancer: a systematic review of reviews. Work.

[16] Chan RJ, Cooper B, Koczwara B, Chan A, Tan CJ, Paul SM, Dunn LB, Conley YP, Kober KM, Levine JD, Miaskowski C (2020). A longitudinal analysis of phenotypic and symptom characteristics associated with inter-individual variability in employment interference in patients with breast cancer. Support Care Cancer.

[17] Miaskowski C, Cooper B, Paul SM, West C, Langford D, Levine JD, Abrams G, Hamolsky D, Dunn L, Dodd M, Neuhaus J, Baggott C, Dhruva A, Schmidt B, Cataldo J, Merriman J, Aouizerat BE (2012). Identification of patient subgroups and risk factors for persistent breast pain following breast cancer surgery. J Pain.

[18] Van Onselen C, Paul SM, Lee K, Dunn L, Aouizerat BE, West C, Dodd M, Cooper B, Miaskowski C (2013). Trajectories of sleep disturbance and daytime sleepiness in women before and after surgery for breast cancer. J Pain Symptom Manag.

[19] McCann B, Miaskowski C, Koetters T, Baggott C, West C, Levine JD, Elboim C, Abrams G, Hamolsky D, Dunn L, Rugo H, Dodd M, Paul SM, Neuhaus J, Cooper B, Schmidt B, Langford D, Cataldo J, Aouizerat BE (2012). Associations between pro- and anti-inflammatory cytokine genes and breast pain in women prior to breast cancer surgery. J Pain.

[20] Chan R, Cooper B, Paul S, Conley Y, Koczwara B, Chan A, et al. Distinct financial distress profiles in patients with breast cancer prior to and for 12 months following surgery. BMJ Support Palliat Care. 2020, In press, bmjspcare-2020-002461. 10.1136/bmjspcare-2020-002461. 10.1136/bmjspcare-2020-002461 32913003

[21] Karnofsky D (1977). Performance scale.

[22] Sangha O, Stucki G, Liang MH, Fossel AH, Katz JN (2003). The self-administered comorbidity questionnaire: a new method to assess comorbidity for clinical and health services research. Arthritis Rheum.

[23] Ferrell BR, Wisdom C, Wenzl C (1989). Quality of life as an outcome variable in the management of cancer pain. Cancer.

[24] Altice CK, Banegas MP, Tucker-Seeley RD, Yabroff KR (2016). Financial hardships experienced by cancer survivors: a systematic review. JNCI.

[25] Spielberger CG, Gorsuch RL, Suchene R, Vagg PR, Jacobs GA (1983). Manual for the state-anxiety (form Y): self evaluation questionnaire.

[26] Bieling PJ, Antony MM, Swinson RP (1998). The state-trait anxiety inventory, trait version: structure and content re-examined. Behav Res Ther.

[27] Kennedy BL, Schwab JJ, Morris RL, Beldia G (2001). Assessment of state and trait anxiety in subjects with anxiety and depressive disorders. Psychiatr Q.

[28] Carpenter JS, Andrykowski MA, Wilson J, Hall LA, Rayens MK, Sachs B, Cunningham LL (1998). Psychometrics for two short forms of the Center for Epidemiologic Studies-Depression Scale. Issues Ment Health Nurs.

[29] Radloff LS (1977). The CES-D scale: a self-report depression scale for research in the general population. Appl Psychol Meas.

[30] Sheehan TJ, Fifield J, Reisine S, Tennen H (1995). The measurement structure of the Center for Epidemiologic Studies Depression Scale. J Pers Assess.

[31] Lee KA, Hicks G, Nino-Murcia G (1991). Validity and reliability of a scale to assess fatigue. Psychiatry Res.

[32] Dhruva A, Dodd M, Paul SM, Cooper BA, Lee K, West C, Aouizerat BE, Swift PS, Wara W, Miaskowski C (2010). Trajectories of fatigue in patients with breast cancer before, during, and after radiation therapy. Cancer Nurs.

[33] Cimprich B, Visovatti M, Ronis DL (2011). The attentional function index--a self-report cognitive measure. Psychooncology.

[34] Cimprich B (1992). Attentional fatigue following breast cancer surgery. Res Nurs Health.

[35] Cimprich B, Ronis DL, Trask C (2005). Pre-treatment factors related to cognitive functioning in women newly diagnosed with breast cancer. Psychooncology.

[36] Jansen CE, Dodd MJ, Miaskowski CA, Dowling GA, Kramer J (2008). Preliminary results of a longitudinal study of changes in cognitive function in breast cancer patients undergoing chemotherapy with doxorubicin and cyclophosphamide. Psychooncology.

[37] Fletcher BS, Paul SM, Dodd MJ, Schumacher K, West C, Cooper B, Lee K, Aouizerat B, Swift P, Wara W, Miaskowski CA (2008). Prevalence, severity, and impact of symptoms on female family caregivers of patients at the initiation of radiation therapy for prostate cancer. J Clin Oncol.

[38] Lee KA (1992). Self-reported sleep disturbances in employed women. Sleep.

[39] Lee KA, DeJoseph JF (1992). Sleep disturbances, vitality, and fatigue among a select group of employed childbearing women. Birth.

[40] Padilla GV, Ferrell B, Grant MM, Rhiner M (1990). Defining the content domain of quality of life for Cancer-patients with pain. Cancer Nurs.

[41] Padilla GV, Presant C, Grant MM, Metter G, Lipsett J, Heide F (1983). Quality of life index for patients with cancer. Res Nurs Health.

[42] Langford DJ, Cooper B, Paul S, Humphreys J, Hammer MJ, Levine J, Conley YP, Wright F, Dunn LB, Miaskowski C (2020). Distinct stress profiles among oncology patients undergoing chemotherapy. J Pain Symptom Manag.

[43] Nylund KL, Asparoutiov T, Muthen BO (2007). Deciding on the number of classes in latent class analysis and growth mixture modeling: a Monte Carlo simulation study. Struct Equ Model.

[44] Muthen BO (2001). Latent variable mixture modeling. In: new developments and techniques in structural equation modeling. Edn. Edited by Marcoulides GA, Schumacher RE.

[45] Muthen LK, Muthen BO (1998). Mplus User's guide (8th ed.), 8th edn.

[46] Wang L, Hong BY, Kennedy SA, Chang Y, Hong CJ, Craigie S, Kwon HY, Romerosa B, Couban RJ, Reid S, Khan JS, McGillion M, Blinder V, Busse JW (2018). Predictors of unemployment after breast cancer surgery: a systematic review and meta-analysis of observational studies. J Clin Oncol.

[47] Ekenga CC, Perez M, Margenthaler JA, Jeffe DB (2018). Early-stage breast cancer and employment participation after 2 years of follow-up: a comparison with age-matched controls. Cancer.

[48] Duijts SF, van Egmond MP, Spelten E, van Muijen P, Anema JR, van der Beek AJ (2014). Physical and psychosocial problems in cancer survivors beyond return to work: a systematic review. Psychooncology.

[49] de Boer AG, Taskila TK, Tamminga SJ, Feuerstein M, Frings-Dresen MH, Verbeek JH (2015). Interventions to enhance return-to-work for cancer patients. Cochrane Database Syst Rev.

[50] Alfano CM, Kent EE, Padgett LS, Grimes M, de Moor JS (2017). Making cancer rehabilitation services work for cancer patients: recommendations for research and practice to improve employment outcomes. PM R.

[51] Bijker R, Duijts SFA, Smith SN, de Wildt-Liesveld R, Anema JR, Regeer BJ (2018). Functional impairments and work-related outcomes in breast cancer survivors: a systematic review. J Occup Rehabil.

[52] Peterman AH, Fitchett G, Brady MJ, Hernandez L, Cella D (2002). Measuring spiritual well-being in people with cancer: the functional assessment of chronic illness therapy--spiritual well-being scale (FACIT-Sp). Ann Behav Med.

[53] Taylor RJ, Chatters LM, Joe S (2011). Religious involvement and suicidal behavior among African Americans and black Caribbeans. J Nerv Ment Dis.

