# Supplementary material for: Distinct employment interference profiles in patients with breast cancer prior to and for 12 months following surgery
Source: BMC Cancer. 2021 Aug 2;21:883. doi: 10.1186/s12885-021-08583-0 (PMC8327049; doi:10.1186/s12885-021-08583-0)
Supplement: Supplementary file 1 — Additional file 1. [file 12885_2021_8583_MOESM1_ESM.docx]

DISTINCT EMPLOYMENT INTERFERENCE PROFILES IN PATIENTS WITH BREAST CANCER PRIOR TO AND FOR 12 MONTHS FOLLOWING SURGERY

Raymond Javan Chan, RN, PhD^1,2,3^

Bruce Cooper, PhD^4^

Louisa Gordon, MPH, PhD^1,5^

Nicolas Hart, PhD^1,2^

Chia Jie Tan, BPharm (Hons)^8,9^

Bogda Koczwara, BMBS FRACP^6^

Kord M. Kober, PhD^4^

Alexandre Chan, Pharm D^7^

Yvette P. Conley, PhD^10^

Steven M. Paul, PhD^4^

Christine Miaskowski, RN, RN, PhD^4^

^1^School of Nursing, Queensland University of Technology, Kelvin Grove, Australia

^2^Cancer and Palliative Care Outcomes Centre, Queensland University of Technology, Kelvin Grove, QLD, Australia

^3^Princess Alexandra Hospital, Metro South Hospital and Health Services, Woolloongabba, Queensland, Australia

^4^School of Nursing, University of California, San Francisco, San Francisco, CA, USA

^5^QIMR Berghofer Medical Research Institute, Queensland Australia

^6^Flinders Centre for Innovation in Cancer, Flinders Medical Centre and Flinders University, Bedford Park, Australia

^7^Department of Clinical Pharmacy Practice, University of California, Irvine, Irvine, CA, USA

^8^Department of Pharmacy, Faculty of Science, National University of Singapore

^9^Department of Pharmacy, National Cancer Centre, Singapore

^10^School of Nursing, University of Pittsburgh, Pittsburgh, PA, USA

Additional File 1. Differences in modifiable and non-modifiable risk factors associated with employment interference based on the findings from a hierarchical linear modeling analysis^a^ and the current latent profile analysis

| **Risk factors** | **HLM Analysis** | **LPA - Differences between**  **High vs None Subgroups** | **LPA: Differences between Low vs None Subgroups** | **LPA: Differences between High vs Low Subgroups** |
| --- | --- | --- | --- | --- |
| Non-modifiable | - Younger age - Lower annual household income - Receipt of an ALND - Receipt of SLNB - Receipt of adjuvant CTX (Time varying covariate) - Receipt of complementary or alternative therapy (Time varying covariate) - Receipt of re-excision or mastectomy of the affected breast (Time varying covariate) | - Younger age - Higher level of education - Being Non-White - Less likely to have gone through menopausal prior to surgery - More advanced stage of disease - Receipt of ALND - Receipt of neoadjuvant CTX - Receipt of adjuvant CTX over the 12 months - Receipt of complementary or alternative therapy over the 12 months - Receipt of re-excision or mastectomy of the affected breast over the 12 months | - Younger age - Higher level of education - Lower comorbidity burden (SCQ score) - More likely to be currently working for pay - Higher annual household income - Receipt of complementary or alternative therapy over the 12 months | - Younger age - Being Non-White - Less likely to be currently working for pay - Lower annual household income - Less likely to have gone through menopausal prior to surgery - More advanced stage of disease - Receipt of ALND - Receipt of neoadjuvant CTX - Receipt of adjuvant CTX over the 12 months - Receipt of re-excision or mastectomy of the affected breast over the 12 months |
| Potentially modifiable | - Higher level of sleep disturbance score (GSDS score) | - Lower functional status (KPS score) - Higher level of trait anxiety (STAI score) - Higher level of state anxiety (STAI score) - Higher level of depressive symptoms (CES-D score) - Higher level of fatigue (LFS score) - Lower level of attentional function (AFI score) - Higher level of sleep disturbance (GSDS score) | - Higher level of fatigue (LFS score) |  |

Abbreviations: AFI = Attentional Function Index, ALND = axillary lymph node dissection, BMI = body mass index, CES-D = Center of Epidemiologic Studies-Depression Scale, CTX = chemotherapy, EI= employment interference, GSDS = General Sleep Disturbance Scale, HLM = hierarchical linear modeling, KPS = Karnofsky Performance Status, LFS = Lee Fatigue Scale, LPA = latent profile analysis, SCQ = Self-administered Comorbidity Questionnaire score, SLNB = sentinel lymph node biopsy, STAI = Spielberger State-Trait Anxiety Inventories

^a^ Chan R, Cooper B, Koczwara B, Chan A, CJ Tan, Paul S, Dunn L, Coley Y, Levine JD, Miaskowski C (2020). A longitudinal study of phenotypic and symptom characteristics associated with inter-individual variability in employment interference in patients with breast cancer. *Supportive Care in Cancer*. Inpress. doi: 10.1007/s00520-020-05312-4
